# Viral Loads Within 6 Weeks After Diagnosis of HIV Infection in Early and Later Stages: Observational Study Using National Surveillance Data

**DOI:** 10.2196/10770

**Published:** 2018-11-05

**Authors:** Richard M Selik, Laurie Linley

**Affiliations:** 1 Division of HIV/AIDS and Prevention National Center for HIV/AIDS, Viral Hepatitis, STD, and TB Prevention Centers for Disease Control and Prevention Atlanta, GA United States

**Keywords:** acute HIV infection, early HIV infection, primary HIV infection, HIV testing, viral load

## Abstract

**Background:**

Early (including acute) HIV infection is associated with viral loads higher than those in later stages.

**Objective:**

This study aimed to examine the association between acute infection and viral loads near the time of diagnosis using data reported to the US National HIV Surveillance System.

**Methods:**

We analyzed data on infections diagnosed in 2012-2016 and reported through December 2017. Diagnosis and staging were based on the 2014 US surveillance case definition for HIV infection. We divided early HIV-1 infection (stage 0) into two subcategories. Subcategory 0α: a negative or indeterminate HIV-1 antibody test was ≤60 days after the first confirmed positive HIV-1 test or a negative or indeterminate antibody test or qualitative HIV-1 nucleic acid test (NAT) was ≤180 days before the first positive test, the latter being a NAT or detectable viral load. Subcategory 0β: a negative or indeterminate antibody or qualitative NAT was ≤180 days before the first positive test, the latter being an HIV antibody or antigen/antibody test. We compared median earliest viral loads for each stage and subcategory in each of the first 6 weeks after diagnosis using only the earliest viral load for each individual.

**Results:**

Of 203,392 infections, 56.69% (115,297/203,392) were reported with a quantified earliest viral load within 6 weeks after diagnosis and criteria sufficient to determine the stage at diagnosis. Among 5081 infections at stage 0, the median earliest viral load fell from 694,000 copies/mL in week 1 to 125,022 in week 2 and 43,473 by week 6. Among 30,910 infections in stage 1, the median earliest viral load ranged 15,412-17,495. Among 42,784 infections in stage 2, the median viral load declined from 44,973 in week 1 to 38,497 in week 6. Among 36,522 infections in stage 3 (AIDS), the median viral load dropped from 205,862 in week 1 to 119,000 in week 6. The median earliest viral load in stage 0 subcategory 0α fell from 1,344,590 copies/mL in week 1 to 362,467 in week 2 and 47,320 in week 6, while that in subcategory 0β was 70,114 copies/mL in week 1 and then 32,033 to 44,067 in weeks 2-6. The median viral load in subcategory 0α was higher than that in subcategory 0β in each of the first 6 weeks after diagnosis (*P*<.001).

**Conclusions:**

In the 1st week after diagnosis, viral loads in early infections are generally several times higher than those in later stages at diagnosis. By the 3rd week, however, most are lower than those in stage 3. High viral loads in early infection are much more common in subcategory 0α than in subcategory 0β, consistent with 0α comprising mostly acute infections and 0β comprising mostly postacute early infections. These findings may inform the prioritization of interventions for prevention.

## Introduction

The 2014 revision of the US surveillance case definition for HIV infection added “stage 0” to its staging system to represent early infection (assumed to last about 6 months after the start of infection). HIV infections are classified in stage 0 if they have evidence of being early—negative or indeterminate HIV test results near the time of diagnosis. Otherwise, they are classified in the later stages—1, 2, or 3 (acquired immunodeficiency syndrome [AIDS]) [1]. Prompt recognition of infections in stage 0 can provide a critical opportunity to prevent transmission of HIV infection during acute (or primary) infection (part of stage 0). Acute infection is often associated with very high viral loads [2-7], which increase the risk of transmission [8]. Intervention would include antiretroviral treatment to suppress the viral load and the provision of “partner services,” in which public health workers interview the patient to identify sex or needle-sharing partners in the past 12 months; locate the partners; and offer them HIV testing, counseling, and linkage to care, as appropriate [9,10]. If infected, such partners may also have early infection.

This analysis was intended to document the high viral loads that justify giving priority to stage 0 infections for intervention to prevent transmission. For that purpose, we compared median earliest viral loads by week in the first 6 weeks after diagnosis for each stage (0, 1, 2, or 3). Another objective was to demonstrate how HIV surveillance data could be used to distinguish between acute infections and other early HIV infections in stage 0, as the highest priority for intervention should be given to acute infections (expected to have the highest viral loads) rather than given equally to postacute early infections (with lower viral loads). To do that, we defined subcategories of stage 0 that approximate acute infection and postacute early infection and compared median viral loads among these two subcategories.

## Methods

### Data

We used data for the 203,392 HIV infections diagnosed during 2012-2016 and reported to the National HIV Surveillance System (NHSS) of the Centers for Disease Control and Prevention through December 2017 from the 50 US states, the District of Columbia, Puerto Rico, and the US Virgin Islands. In the software of the NHSS database, the stage at diagnosis can be automatically classified as stage 0 only for HIV infections diagnosed in or after 2014, when the definition of stage 0 was published [1]. For this analysis, however, we retroactively extended the application of the definition of stage 0 to infections diagnosed in 2012 and 2013 to increase the number of stage 0 diagnoses available for analysis.

We excluded the 24.03% (48,880/203,392) of infections for which the stage at diagnosis could not be determined. Their reported data did not include the negative or indeterminate HIV test results required to meet the criteria for stage 0 nor a CD4 T-lymphocyte test result or opportunistic illness diagnosis required to meet the criteria for stage 1, 2, or 3 within 3 months after diagnosis.

We assumed that specimens for earliest viral loads would generally have been collected before starting antiretroviral therapy to enable physicians to assess the effect of therapy by comparing subsequent viral loads with the baseline viral load. Therefore, to reveal the natural trend of viral loads before their suppression by antiretroviral drugs, we restricted our analysis to the viral load with the earliest specimen collection date within 6 weeks after diagnosis for each infection. This restriction removed another 32,047 infections from the analysis because they had no viral load within 6 weeks after diagnosis. The date when antiretroviral drugs were first received was reported for only 17.27% (21,157/122,465) of the remaining infections, but it preceded the date of the first viral load for 10.04% (2,125/21,157) of them. Therefore, we also excluded those cases in which the drug was known to have preceded the viral load to minimize the effects of antiretroviral drugs on the viral loads.

To try to avoid erroneous data on earliest viral loads, we excluded another 4711 cases in which the first viral load was reported to be undetectable or 0-19 copies/mL. Such low values would be unlikely in the absence of antiretroviral prophylaxis or therapy started on the basis of a diagnosis earlier than the date of the reported first positive HIV test. In addition, viral loads reported as 0-9 copies/mL may actually have been logarithmically transformed values that could not be compared with the untransformed values on which our analysis was based. Enumerated viral loads reported to be undetectable probably represented the lower limit of the test’s ability to quantify the viral load rather than its actual value. We also excluded another 167 cases with viral loads for which no numerical value was reported. To calculate the number of days between the diagnosis date and viral load date accurately, we also excluded 165 cases in which data for one or both of these dates were incomplete (eg, missing the day component). The final analytic file had data on 115,297 HIV infections (each corresponding to the first viral load within 6 weeks after a diagnosis), representing 56.68% (115,297/203,392) of the total reported cases diagnosed in 2012-2017.

### Definitions

#### Stage 0

**Figure 1:**
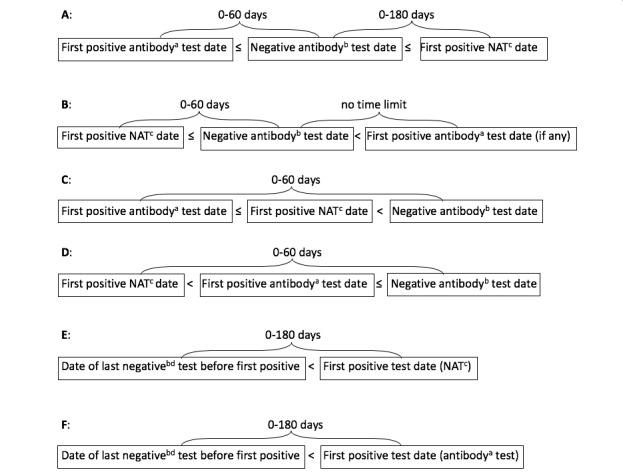
Test sequences defining stage 0 preliminary subgroups. Superscripts are as follows: a) Positive “antibody” test results include positive results from the antigen component or the antibody component of combination antigen or antibody tests. b) “Negative antibody” test results include indeterminate results from supplemental immunoglobulin G–only supplemental antibody tests and negative results from antigen or antibody tests in which only the antibody component is negative or in which both the antigen and antibody components are negative. c) Positive nucleic acid tests (NATs) include qualitative and quantitative (viral load) tests. d) Negative NATs include only qualitative NATs.

The HIV surveillance case definition published in 2014 says that stage 0 may be recognized based on either testing history—“a negative or indeterminate HIV test…result within 180 days before the first confirmed positive HIV test result”—or a testing algorithm—“a sequence of tests performed as part of a laboratory testing algorithm that demonstrate the presence of HIV-specific viral markers such as…nucleic acid (RNA or DNA) 0-180 days before or after an antibody test that had a negative or indeterminate result” [1]. Unfortunately, this definition is impractical to apply strictly to NHSS data because our data do not state whether multiple reported tests belong to the same diagnostic algorithm or are from unrelated testing events. Therefore, the definition of stage 0 used for our analysis of NHSS data is based only on the sequence of HIV test results, and not on whether they were intended to constitute a diagnostic algorithm. We classified the stage of disease at diagnosis as stage 0 based on any of the following possible sequences of positive (or reactive) and negative (or nonreactive) or indeterminate HIV test results (as shown in Figure 1):

- (1) The first positive HIV test result was from an antibody or antigen/antibody test; (2) it was accompanied (on the same date) or followed within ≤60 days by a negative or indeterminate result from an HIV antibody test or the antibody component of a combination antigen/antibody test; and (3) a positive HIV-1 nucleic acid test (NAT, qualitative or viral load) result was within ≤180 days after (or on the same date as) the negative or indeterminate test.
- (1) The first positive HIV test result was an HIV-1 NAT (and there was no positive antibody test on the same date) and (2) the NAT was accompanied or followed within ≤60 days by a negative or indeterminate result from an HIV antibody test or the antibody component of a combination antigen/antibody test (regardless of whether there was a later positive antibody test).
- (1) The first positive HIV test result was from an antibody or antigen/antibody test; (2) it was accompanied or followed by a positive NAT; and (3) both positive tests were followed by (were not on the same date as) a negative or indeterminate result from an HIV antibody test or the antibody component of a combination antigen/antibody test that was within ≤60 days after the first positive test.
- (1) The first positive HIV test result was from an HIV-1 NAT; (2) it was followed by (was not on the same date as) a positive result from an HIV antibody or antigen/antibody test; and (3) both positive tests were followed by a negative or indeterminate result from an HIV antibody test or the antibody component of a combination antigen/antibody test that was within ≤60 days after (not on the same date as) the first positive test (NAT) but that could have been on the same date as the second positive test (the antibody or antigen/antibody test).
- (1) A negative result from an HIV-1 qualitative NAT or a negative or indeterminate result from an HIV antibody or antigen/antibody test was followed within ≤180 days by, and was not on the same date as, (2) the first positive HIV test result, which was from an HIV-1 NAT.
- (1) A negative result from an HIV-1 qualitative NAT or a negative or indeterminate result from an HIV antibody or antigen/antibody test was followed within ≤180 days by, and was not on the same date as, (2) the first positive HIV test result, which was from an HIV antibody or antigen test that was confirmed by (3) a positive result from a second (supplemental) HIV test of a different (orthogonal) type.

We defined preliminary subcategories of stage 0 (0A through 0F) based on each of the above sequences (A through F). Subcategory 0A includes infections recognized as acute based on results from a testing algorithm recommended by the Association of Public Health Laboratories and the US Centers for Disease Control and Prevention [11]. However, it also includes a small proportion of sequences that did not conform exactly to recommendations. For example, in a small percentage of those, the first positive result was from an antibody test that could detect only immunoglobulin G antibody, which would have been more appropriate as a supplemental test rather than the initial test. In others, the negative test result was from an antigen/antibody test that could detect both immunoglobulin M and immunoglobulin G antibodies, which would have been more appropriate as an initial test. Subcategory 0B includes infections recognized as acute based on results from a testing algorithm recommended for populations with a high incidence of HIV infection, in which a specimen for a pooled NAT is collected on the same date as the specimen for an initial HIV antibody immunoassay that had a negative result [12].

Subcategories 0C and 0D would have been the same as 0A and 0B, respectively, except that the negative or indeterminate antibody or antigen/antibody test follows both the positive NAT and the positive antibody or antigen/antibody test rather than preceding or being between them (Figure 1). Subcategories 0C and 0D seem not to fit the criteria for stage 0 in the published case definition [1] because their test sequences do not conform to any recommended algorithm and they do not fit a testing history of a negative result within 180 days before the first positive result. We included them in stage 0 for this analysis because we found them to be associated with high viral loads characteristic of acute infection.

Subcategories 0A through 0D are mutually exclusive, but their test sequences could overlap those of subcategories 0E or 0F in some cases. If there was such an overlap, we classified the cases in subcategories 0A through 0D rather than in 0E or 0F because the former are based on a more recent negative test result than the latter and therefore are more likely to represent acute infection. Subcategories 0E and 0F differ only by the fact that the first positive test in 0E was a NAT, while the first positive test in 0F was an antibody or antigen/antibody test. Since the interval between the negative or indeterminate test and the first positive test in 0E or 0F could be up to 180 days, these two tests would generally not belong to the same testing algorithm and most likely represent two separate testing events, of which the first received the interpretation that HIV infection was absent and the second that HIV was present.

We considered subcategories 0A through 0F as “preliminary” because, after our preliminary analysis showed that subcategories 0A through 0E were associated with high viral loads soon after diagnosis (Tables 3-5), we combined subcategories 0A through 0E into a larger subcategory named “0α” (assumed to approximate acute infection) and named the remainder (subcategory 0F) “0β” (assumed to consist mostly of postacute early infection).

For our analysis, an undetectable viral load before the first positive test result was not accepted as the negative HIV test result indicative of the earliness of the infection in subcategories 0E or 0F. An unpublished investigation (personal communication from Galang RR and Peters PJ, December 2014) found that such undetectable viral loads were not reliable evidence of early infection but were instead often due to therapy for established infections diagnosed on the basis of earlier test results that had not been reported to the surveillance system.

We did not classify the stage at diagnosis as stage 0 if a reported test result contradicted the first positive HIV test result or the negative or indeterminate HIV test result that would have been the indicator of earliness (ie, the contradictory test and the test that it contradicted were on same date and of the same type but had opposite results) because such contradictory results imply that one of them was erroneous. This happened in 10.4% (844/8077) of infections that would otherwise have met the criteria for stage 0 (including some that were excluded from our analysis for other reasons, eg, not having a reported viral load within 6 weeks after diagnosis); these were instead classified in other stages (1, 2, or 3) and kept in the analysis unless removed for other reasons described above.

The criteria for stage 0 took precedence over criteria for more advanced stages. If the criteria for stage 0 were not met, the stage at diagnosis was defined by the earliest criteria for stage 1, 2, or 3 met within 3 months after diagnosis of HIV infection. These criteria were based on a CD4 T-lymphocyte count or percentage indicative of stage 1, 2, or 3, or diagnosis of an opportunistic illness indicative of stage 3 [1]. If earliest criteria were met for different stages (other than stage 0) in the same month, the stage at diagnosis was selected as the most advanced of those stages.

#### Test Date

We defined a test date as the date on which the test specimen was collected. This could pertain to the dates of the positive and negative tests used for diagnosis of stage 0 or the date of the earliest viral load after or on the same date as the diagnostic tests. In some cases, the first viral load could function as a diagnostic test.

#### Diagnosis Date for HIV Infection

We defined the “diagnosis date” as the earliest date of objective evidence of HIV infection, selected from the earliest of the following 4 possible events: (1) the first positive HIV test (this was the earliest objective evidence for 96% of diagnoses; rarely was it several days earlier than the confirmatory test date in a multitest algorithm); (2) the first diagnosis of an opportunistic illness indicative of stage 3; (3) the first CD4 T-lymphocyte count or percentage low enough to indicate stage 3; or (4) the first “clinical” diagnosis of HIV infection documented in a medical record but for which a prior positive HIV test result on which the diagnosis was based could not be found by surveillance staff (accounting for <1% of diagnoses). It should be borne in mind that, for infections not diagnosed in stage 0, the diagnosis date may be years after infection began, particularly for infections diagnosed in stage 3. Among all infections diagnosed in 2015, the estimated median interval from infection to diagnosis was 3 years [13].

### Statistical Methods

Viral load data for each patient were unavailable for each week after diagnosis, as each individual was observed at only one point in time (the date of his earliest viral load) in whichever week it occurred. In aggregate, however, grouped by week after diagnosis, we assumed that these data would simulate a longitudinal series of weekly viral loads representative of what would have occurred in the average person in the study population if that individual had been followed over time. We calculated the median, 25th percentile, and 75th percentile for the earliest viral loads by week after diagnosis for each stage of disease at diagnosis and for each subcategory of stage 0. To assess the possible effect of unreported antiretroviral drugs on the speed of the decline in the viral load by week after diagnosis among all infections diagnosed in stage 0, we compared results for viral loads that had missing information on when antiretroviral drugs were started with results for viral loads reported to have been on or before the date when antiretroviral drugs were started (assumed not to have been affected by such drugs).

To test the statistical significance of differences between median viral loads in two different stages, we did pairwise two-sample Wilcoxon comparisons using PROC NPAR1WAY in SAS software, version 9.4 for Windows (SAS Institute, Inc, Cary, NC, USA). We used the Dwass, Steel, Critchlow-Fligner option to generate multiple comparisons (eg, 6 combinations of pairs from 4 categories [stages 0, 1, 2, and 3], or 10 combinations of pairs from 5 categories [stage-0 subcategories 0α and 0β and stages 1, 2, and 3]) for each family of comparisons (one family per week) [14]. We used Holm’s method for stepwise adjustment of the significance threshold for each comparison to account for the number of comparisons in each family [15,16]. This adjustment was needed only if the *P* value was <.05 but ≥.001.

This analysis did not require approval by an institutional review board because it used only data reported to the NHSS by state or local public agencies as part of routine public health notifiable disease surveillance of HIV infection. The Centers for Disease Control and Prevention has determined that the collection of these data is not research as it is intended primarily for the purpose of disease control. In addition, the dissemination of the results of this analysis could not have any adverse effect on the subjects to whom the data pertain because the tabulations do not identify individuals.

## Results

**Figure 2:**
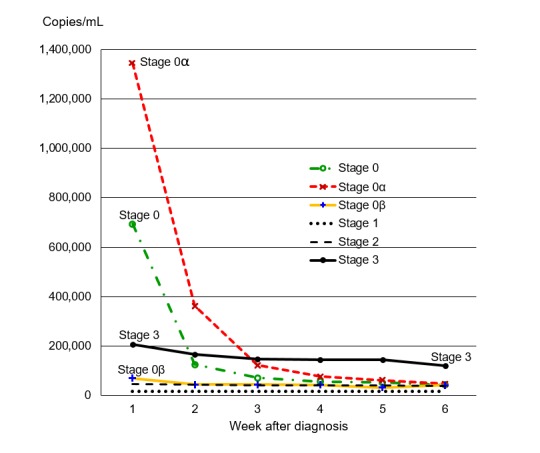
Median earliest viral load (in copies/mL) by week after diagnosis and stage of disease at diagnosis of HIV infection, comparing stages 0, 1, 2, and 3 and stage 0 subcategories 0α (0A+0B+0C+0D+0E) and 0β (0F).

Among the 115,297 infections that remained in our analysis after we applied the exclusion criteria described above, the stage of disease at diagnosis was stage 0 for 4.40% (5081/115,297), stage 1 for 26.80% (30,910/115,297), stage 2 for 37.10% (42,784/115,297), and stage 3 for 31.70% (36,522/115,297). Among the infections in stage 0, the median earliest viral load fell from 694,000 copies/mL in week 1 to 125,022 in week 2 and 43,473 by week 6 (Table 1; Figure 2). In stage 1, the median earliest viral load alternated weekly between increasing and decreasing, ranging from a high of 17,495 copies/mL in week 2 to a low of 15,412 in week 5 (Table 1). In stage 2, the viral load declined from 44,973 copies/mL in week 1 to 38,497 in week 6 (Table 2). In stage 3, the viral load dropped from 205,862 copies/mL in week 1 to 167,297 in week 2 and 119,000 by week 6 (Table 2). In week 1, the median earliest viral load for diagnoses in stage 0 was much higher than that for diagnoses in stages 1, 2, or 3 (*P*<.001 for comparison of each pair of results), but by week 2, it did not differ significantly from that for diagnoses in stage 3 (*P*=.05), and by week 3, it had fallen below that for diagnoses in stage 3 (*P*<.001). The median viral load for diagnoses in stage 0 was higher than that for diagnoses in stage 2 in weeks 1-4 (*P*<.001) but did not differ from it in week 5 or 6 (*P*>.58).

**Table 1:** Median earliest viral load (in copies/mL) by week after diagnosis and stage of disease at diagnosis of HIV infection, for stages 0 and 1. (See Table 2 to compare with stages 2 and 3.)

Week	Stage 0			Stage 1		
	N	Median	25th-75th percentiles	N	Median	25th-75th percentiles
1^st^	2635	694,000	82,510-5,177,075	12,214	16,219	2,235-67,608
2^nd^	934	125,022	26,920-857,030	6476	17,495	3,335-55,845
3^rd^	629	70,886	16,113-289,407	4694	15,694	3,310-48,100
4^th^	408	55,734	16,898-210,577	3363	16,500	3,690-50,734
5^th^	277	52,067	10,500-168,526	2418	15,412	3,100-45,774
6^th^	198	43,473	11,890-119,960	1745	16,649	3,347-46,236

**Table 2:** Median earliest viral load (in copies/mL) by week after diagnosis and stage of disease at diagnosis of HIV infection, for stages 2 and 3. See Table 1 to compare with stages 0 and 1.

Week	Stage 2				Stage 3		
	N	Median	25^th^-75^th^ percentiles	N		Median	25^th^-75^th^ percentiles
1^st^	17,098	44,973	13,200-137,765	20,094		205,862	75,700-537,471
2^nd^	8968	42,892	14,400-107,936	6234		167,297	63,386-456,000
3^rd^	6357	39,800	13,994-99,185	4091		146,700	55,150-393,724
4^th^	4523	40,045	13,670-96,817	2726		144,840	53,085-405,503
5^th^	3326	41,549	14,544-101,780	1932		143,560	49,038-386,008
6^th^	2512	38,497	14,370-86,818	1445		119,000	44,620-359,040

**Table 3:** Median earliest viral load (in copies/mL) by week after diagnosis and stage of disease at diagnosis of HIV infection, for stage 0 preliminary subcategories 0A and 0B.

Week	Subcategory 0A			Subcategory 0B		
	N	Median	25^th^-75^th^ percentiles	N	Median	25^th^-75^th^ percentiles
1^st^	1,083	1,258,925	238,487-7,413,102	408	1,307,793	210,000-9,768,809
2^nd^	426	338,888	46,539-1,610,000	68	350,482	55,646-2,184,015
3^rd^-6^th^	715	77,380	16,847-299,330	89	104,500	32,451-347,555

**Table 4:** Median earliest viral load (in copies/mL) by week after diagnosis and stage of disease at diagnosis of HIV infection, for stage 0 preliminary subcategories 0C and 0D.

Week	Subcategory 0C			Subcategory 0D			
	N	Median	25th-75th percentiles		N	Median	25th-75th percentiles
1^st^	187	1,780,000	456,000-7,500,000		125	2,160,000	106,000-8,426,138
2^nd^	76	635,690	77,402-4,393,963		11	1,778,279	155,054-4,579,249
3^rd^-6^th^	71	75,300	11,000-550,398		24	64,294	22,292-237,806

**Table 5:** Median earliest viral load (in copies/mL) by week after diagnosis and stage of disease at diagnosis of HIV infection, for stage 0 preliminary subcategories 0E and 0F.

Week	Subcategory 0E			Subcategory 0F		
	N	Median	25th-75th percentiles	N	Median	25^th^-75^th^ percentiles
1st	224	1,346,776	139,122-5,934,603	608	70,114	11,233-271,000
2nd	17	294,798	60,656-756,500	336	44,467	8,456-125,087
3rd-6th	16	135,540	46,915-530,205	597	42,000	11,040-114,000

In the first week after diagnosis, infections in stage 0 preliminary subcategories 0A, 0B, 0C, 0D, and 0E had median viral loads exceeding 1.2 million copies/mL compared with only 70,114 copies/mL for infections in subcategory 0F (Tables 3-5). In week 2, median viral loads for subcategories 0A through 0E remained higher (>290,000 copies/mL) than the 44,467 copies/mL for subcategory 0F, as well as higher than the median viral loads for stages 1 (17,495), 2 (42,892), and 3 (167,297; *P*<.001 for each of these comparisons). Subcategories 0E and 0F were both defined by a negative or indeterminate test result within 180 days before the first positive test result (a positive NAT for 0E and a positive antibody test for 0F), but their earliest viral loads in the first 2 weeks after diagnosis differed greatly (Table 5). This difference may be explained in part by the interval between the first positive test result and the preceding negative or indeterminate test result being short (1 or 2 weeks) for a much greater proportion of infections in subcategory 0E (130/257, 51%) than in subcategory 0F (139/1541, 9.0%) and by the median value for this interval being only 9 days for subcategory 0E while being 98 days for subcategory 0F. Due to the small number of observations per week after week 2 for subcategories 0D and 0E, we combined weeks 3 through 6 into a single time period in Tables 3-5. Because of the similarity of findings for preliminary subcategories 0A through 0E evident in Tables 3-5, we combined them into a larger subcategory named “0α” for further analysis by week and renamed subcategory 0F as “0β” (Table 6).

Median viral loads in stage 0 subcategory 0α were higher than those in subcategory 0β in each of the first 4 weeks after diagnosis (*P*<.001 for weeks 1, 2, and 3 and *P*=.008 for week 4, significant compared to a Holm-adjusted threshold of *P*=.02) but did not differ significantly from them in weeks 5 or 6 (*P*>.39; Table 6). The median viral load for stage 0 subcategory 0α was also much higher than that for stage 3 in weeks 1 and 2 (*P*<.001) but did not differ from it in week 3 (*P*=.09) and was lower than that for stage 3 in weeks 4-6 (*P*<.001; Figure 2). Median viral loads for subcategory 0α were higher than those for stage 2 in weeks 1 through 4 (*P*<.001) but did not differ from them in weeks 5 or 6 (*P*>.14). The median viral load for subcategory 0β was greater than that for stage 2 in week 1 (*P*<.001) but did not differ from it in later weeks (*P*>.88). In every week, the median viral load for stage 0 subcategory 0β was greater than that for stage 1, and the median viral loads for stages 1, 2, and 3 differed significantly from one another in the same direction as the order of their names (ie, 1<2<3; *P*<.001).

**Table 6:** Median earliest viral load (in copies/mL) by week after diagnosis and stage of disease at diagnosis of HIV infection, comparing stage 0 subcategory 0α with subcategory 0β.

Week	Subcategory 0α (0A+0B+0C+0D+0E)			Subcategory 0β (0F)				
	N	Median	25th-75th percentiles	N		Median		25th-75th percentiles
1^st^	2027	1,344,590	228,000-7,630,000		608	70,114	11,233-271,000	
2^nd^	598	362,467	50,721-1,905,461		336	44,467	8,456-125,087	
3^rd^	375	122,970	21,600-413,431		254	43,729	9,460-122,825	
4^th^	243	77,100	19,570-301,190		165	42,300	13,900-131,377	
5^th^	177	61,414	12,600-201,000		100	32,033	7,084-112,520	
6^th^	120	47,320	8,628-171,652		78	41,606	19,120-87,670	

Although high viral loads were much more common among infections diagnosed in subcategory 0α than in subcategory 0β or stages 1, 2, or 3, a small percentage of infections in subcategory 0β and other stages did have high viral loads. Earliest viral loads exceeding 500,000 copies/mL occurred in 11.6% (179/1541) of the infections in subcategory 0β, 4.20% (1310/30,910) of those in stage 1, 7.10% (3039/42,784) of those in stage 2, and 24.17% (8831/36,522) of those in stage 3 compared with 50.0% (1754/3540) of those in subcategory 0α. Among infections in subcategory 0β having a viral load of >500,000 copies/mL, the interval between the first positive test and the last prior negative test ranged from 1 to 176 days (with a median of 66 days); it could have been up to 180 days by the definition of 0β.

Among the 3540 infections diagnosed in subcategory 0α, the decline in the median earliest viral loads in the subgroup of 2834 cases with missing information on when antiretroviral drugs were started was similar to that in the subgroup of 706 cases in which antiretroviral drugs were started no earlier than the date on which the viral load specimen was collected. In each of these subgroups, the median earliest viral load decreased by more than half from week 1 to week 2 and did so again from week 2 to week 3. By week 4, the median viral load in each subgroup had decreased by >95% compared with its value for week 1 (from 1,298,413 to 41,687 copies/mL among the 2834 cases with missing antiretroviral information and from 1,485,669 to 73,809 copies/mL among the 706 cases reported to have started antiretroviral drugs no earlier than the viral load date).

## Discussion

### Principal Findings

Our findings confirmed that early HIV infection, represented by stage 0, is associated with viral loads higher than those in infections diagnosed in later stages of the disease. However, this was true mainly in the first week after diagnosis, when the median viral load among infections diagnosed in stage 0 was more than three times than that among infections diagnosed in stage 3.

We also found that stage 0 preliminary subcategories 0A, 0B, 0C, 0D, and 0E, which we combined as subcategory 0α, were associated with viral loads higher than those in preliminary subcategory 0F, which we renamed as subcategory 0β (stage 0 = 0α + 0β). This difference should be expected because most of 0α (preliminary subcategories 0A, 0B, 0C, and 0D) is limited to diagnoses in which a negative or indeterminate antibody test result indicative of the earliness of infection was either on the same date as or ≤60 days after the first positive HIV test date, and the median interval between the first positive HIV test and the last prior negative test was much shorter for infections in preliminary subcategory 0E (9 days) than for those in subcategory 0F (98 days). Thus, a negative or indeterminate test result was closer in time to viral loads in the first week after diagnosis for most infections in subcategory 0α than it was for most infections in subcategory 0β. This allowed most infections in subcategory 0β to have enough time for complete seroconversion and a decline in the viral load before it was first measured. This difference between subcategories 0α and 0β could justify more urgent intervention to suppress the viral load and to provide partner services to prevent transmission if infections are diagnosed in subcategory 0α rather than in subcategory 0β. If so, the NHSS software should be upgraded to distinguish automatically between stage 0 subcategories 0α and 0β to help health departments to account for this difference when they prioritize prevention efforts.

On the other hand, 11.6% (179/1541) of infections in subcategory 0β had viral loads >500,000 copies/mL, suggesting that they might be acute infections. This happened despite an interval of as long as 176 days between the first positive HIV test and the last prior negative test because the infection could have started long after that last negative test and much nearer to the first positive test date. In addition, a small percentage of infections in stages 1 and 2 had viral loads exceeding 500,000 copies/mL. These might actually have been acute infections that were misclassified in stages 1 or 2 because they were missing negative or indeterminate HIV test results required to be classified in stage 0. Therefore, when deciding which infections should receive the highest priority for prevention of transmission, consideration should be given not only to whether the criteria are met for stage 0 subcategory 0α but also to whether other evidence, such as a high viral load near the time of diagnosis, suggests acute infection.

If the restriction of our analysis to each patient’s earliest detectable viral load succeeded in excluding viral loads influenced by antiretroviral drugs, then our findings may accurately reflect the natural trend of viral loads before suppression by drugs. Our success in excluding the influence of antiretroviral drugs is suggested by the similarity of the decline in median earliest viral loads in the subgroup of subcategory 0α in which antiretroviral therapy was reported to have started no earlier than the viral load specimen collection date (when the earliest viral load was assumed not to have been affected by antiretroviral drugs) and those in the larger subgroup with missing information about antiretroviral drugs. It is also consistent with the similarity of the median viral loads we found for subcategory 0α and those found in a cohort of 19 untreated high-risk persons in Thailand [17]. In that cohort, the median viral load peaked at about 2,500,000 copies/mL in week 3 after the diagnosis of acute infection, dropped to 63,000 copies/mL in week 6, and remained at about the same level up to 144 weeks later in the absence of treatment. Similarly, in our study, the median earliest viral loads for subcategory 0α dropped from about 1,250,000 copies/mL in week 1 to about 40,000 copies/mL in week 6 after diagnosis. In contrast, our findings for subcategory 0α differed from the lower viral loads found in a cohort of 71 persons who received antiretroviral therapy promptly after the diagnosis of acute infection, in which the median viral load peaked at about 500,000 copies/mL in week 2 and dropped to about 2,500 copies/mL in week 4 and to <100 copies/mL after week 12 [17]. If our findings reflect viral loads in the absence of antiretroviral therapy, then they imply that the interval after diagnosis of acute HIV infection in which most viral loads exceed 500,000 copies/mL lasts <2 weeks. Thus, antiretroviral therapy to suppress the high viral loads of acute infection may be too late to make much difference if not started very shortly after diagnosis. By the third week after diagnosis, the viral load would probably have spontaneously declined to a level similar to that found in stage 2.

Our analysis was intended to assess the extent to which early infections have extremely high viral loads, as had been reported among acute infections because such high viral loads demand urgent intervention to prevent transmission. However, such intervention in early infection should perhaps receive high priority even after the acute phase, when the viral load has declined to a more stable lower level, as some studies suggest that the viruses in early infections may be more infectious than those in later infections with similar viral loads. This may be due to a partial immune response that develops in older infections, which might favor viruses having mutations that resist the immune response but at the expense of reducing their transmissibility [18-20]. In addition, even if an infection is no longer acute by the time of diagnosis, a diagnosis in stage 0 implies that the infection was recently acute and the viral load was therefore probably very high (even if only briefly), so transmission to recent sex partners would be more likely than if the infection had been diagnosed in a later stage. Therefore, such postacute early infections should also receive high priority for partner services. Another reason to give priority to intervention in stage 0, even after viral loads have declined, is that treatment of HIV infection within 6 months after the start of infection (approximately the time frame of stage 0) could reduce the patient’s risk of morbidity and mortality. Such early treatment is associated with a smaller HIV reservoir size, lower levels of immune activation, and a higher probability of restoration of CD4 T-lymphocyte counts to normal levels [17,21-25].

### Limitations

Our analysis was limited by its dependence on the negative or indeterminate HIV test results needed to meet criteria for stage 0 being reported by health departments to the NHSS database at the Centers for Disease Control and Prevention. Health departments may be unable to report these results to the NHSS if laboratories and health care providers do not report them to health departments. It may also be impossible for health care providers to recognize most early HIV infections because most HIV-infected patients may not present themselves for diagnostic evaluation until after complete seroconversion (when HIV tests would no longer have negative or indeterminate results) and because even patients who arrive during acute HIV infection may not be tested for it until after seroconversion if physicians do not suspect it as its symptoms are nonspecific. Early infections may then be misclassified as later infections, including some as stage 3 (AIDS) because low CD4 T-lymphocyte counts and opportunistic illnesses meeting criteria for stage 3 sometimes occur transiently in acute infection [26,27].

The stage of HIV disease at diagnosis found in our analysis depended on the frequency with which persons were tested for HIV infection, which can be estimated from the interval between HIV tests before diagnosis, based on data collected after diagnosis. Among the 6.94% (14,128/203,392) of all persons reported with HIV infection who had at least one reported negative HIV test result >28 days before diagnosis (implying it was from a prior testing event rather than part of a multitest algorithm on which a diagnosis of acute infection was based), the median interval between successive tests (calculated from the last 1-6 negative tests before diagnosis or between the diagnosis date and the last prior negative test) increased as the stage at diagnosis became more advanced. It rose from 121 days for stage 0, to 406 days for stage 1, to 533 days for stage 2, and to 1,098 days for stage 3 (*P*<.001 Wilcoxon test for each pair of stages compared). The longer intertest intervals, leading to diagnostic delays, may reflect less access to and use of the health care system.

The wide range between the 25th and 75th percentiles of the earliest viral loads for each stage and week after diagnosis (Tables 1 and 2) to some extent may be due to misclassification of the stage of disease “at diagnosis.” Classification in stages 1, 2, or 3 could have been based on a CD4 count specimen obtained up to 3 months (16 weeks if just under 4 months) after diagnosis, during which time the CD4 count level could have changed greatly from what it was on the exact date of diagnosis or the earliest viral load. Among infections diagnosed in stage 0, the wide range of earliest viral loads could have been due in part to differences in the interval between the start of infection and the diagnosis date, during which viral loads could have risen from low to high levels and then fallen back.

Our analysis was also limited by the fact that data from the NHSS are incomplete (eg, missing the day component of some dates, some test results, information about antiretroviral drugs) and sometimes of questionable quality (eg, contradictory test results). We compensated for these limitations in NHSS data by cleaning them in various ways, such as by excluding observations with incomplete dates or supposedly earliest viral loads that were reported to be undetectable or extremely low (0-19 copies/mL) and excluding those cases from stage 0 that had contradictory results (positive and negative) from apparently the same type of test on the same date. Even after the exclusion of these observations, our use of NHSS data brought the advantage of a much larger number of observations than could have been obtained from a study limited to patients receiving care from a small number of providers.

The final study population of 115,297 persons, which accounted for 56.7% of the 203,392 US residents reported with HIV infection diagnosed during 2012-2016, may not have been representative of all US residents because its demographic and other distributions differed significantly (*P*<.001, chi-square test) from those of the 88,095 persons excluded from the study population. In the study population, non-Hispanic whites were more common (32,447/115,297, 28.14% versus 20,534/88,095, 23.30%), non-Hispanic blacks were less common (46,821/115,297, 40.60% versus 41,214/88,095, 46.78%), persons aged >35 years at diagnosis were more common (52,334/115,297, 45.40% versus 36,550/88,095, 41.48%), the proportion residing in the Southern US region was lower (54,419/115,297, 47.19% versus 46,917/88,095, 53.25%), the proportion for which the transmission category was “men who had sex with men” was higher (66,009/115,297, 57.25% versus 46,411/88,095, 52.68%), the proportion reported with no identified HIV risk factor was lower (22,908/115,297, 19.86% versus 20,684/88,095, 23.47%), and the proportion whose infection was diagnosed after 2014 was higher (48,298/115,297, 41.89% versus 32,114/88,095, 36.45%). However, these differences between the included and excluded populations do not mean that our findings about stage-specific trends in viral loads in the first 6 weeks after diagnosis were invalid or that we would not have found similar trends among excluded persons if the missing data on test results and test dates that was the reason for excluding them had been available.

We interpreted the decreasing trends in earliest viral loads by week after diagnosis as if they represented results for individuals followed weekly in the absence of therapy, but actually each individual was observed at only one point in time (the date of his earliest viral load). Longitudinal data on weekly viral loads for individuals who were not receiving antiretroviral drugs would have provided a scientifically sounder basis for analyzing viral load trends during the first several weeks after diagnosis, but such a study would not have been practical or ethically feasible, as current treatment guidelines recommend starting therapy soon after diagnosis [28].

### Conclusions

In summary, we confirmed that viral loads among infections in early infection (stage 0) are generally several times higher than those in later stages at diagnosis, particularly during the first week after diagnosis. By the 4th week, however, they are generally lower than those in stage 3. Viral loads are also higher for subcategory 0α of stage 0 than for subcategory 0β in the first 4 weeks after diagnosis. These findings may be useful in allocating prevention resources by indicating which infections should receive the highest priority for urgent intervention. Where health departments do not have the resources to intervene immediately for all persons with a new diagnosis of HIV infection to ensure linkage to care, counseling to prevent transmission, and partner services, they should give higher priority to those with infections diagnosed in stage 0, especially those in subcategory 0α or known to have recently had a high viral load, and should try to do so within the first 2 weeks after diagnosis.

## Abbreviations

HIV: human immunodeficiency virus
NAT: nucleic acid test
NHSS: National HIV Surveillance System
